# Terpenes Increase the Lipid Dynamics in the *Leishmania* Plasma Membrane at Concentrations Similar to Their IC_50_ Values

**DOI:** 10.1371/journal.pone.0104429

**Published:** 2014-08-07

**Authors:** Heverton Silva Camargos, Rodrigo Alves Moreira, Sebastião Antonio Mendanha, Kelly Souza Fernandes, Miriam Leandro Dorta, Antonio Alonso

**Affiliations:** 1 Instituto de Física, Universidade Federal de Goiás, Goiânia, GO, Brazil; 2 Engenharia Elétrica, Fundação Universidade Federal do Tocantins, Palmas, TO, Brasil; 3 Instituto de Patologia Tropical e Saúde Publica, Departamento de Imunologia e Patologia Geral, Universidade Federal de Goiás, Goiânia, GO, Brazil; Cornell University, United States of America

## Abstract

Although many terpenes have shown antitumor, antibacterial, antifungal, and antiparasitic activity, the mechanism of action is not well established. Electron paramagnetic resonance (EPR) spectroscopy of the spin-labeled 5-doxyl stearic acid revealed remarkable fluidity increases in the plasma membrane of terpene-treated *Leishmania amazonensis* promastigotes. For an antiproliferative activity assay using 5×10^6^ parasites/mL, the sesquiterpene nerolidol and the monoterpenes (+)-limonene, α-terpineol and 1,8-cineole inhibited the growth of the parasites with IC_50_ values of 0.008, 0.549, 0.678 and 4.697 mM, respectively. The IC_50_ values of these terpenes increased as the parasite concentration used in the cytotoxicity assay increased, and this behavior was examined using a theoretical treatment of the experimental data. Cytotoxicity tests with the same parasite concentration as in the EPR experiments revealed a correlation between the IC_50_ values of the terpenes and the concentrations at which they altered the membrane fluidity. In addition, the terpenes induced small amounts of cell lysis (4–9%) at their respective IC_50_ values. For assays with high cell concentrations (2×10^9^ parasites/mL), the incorporation of terpene into the cell membrane was very fast, and the IC_50_ values observed for 24 h and 5 min-incubation periods were not significantly different. Taken together, these results suggest that terpene cytotoxicity is associated with the attack on the plasma membrane of the parasite. The *in vitro* cytotoxicity of nerolidol was similar to that of miltefosine, and nerolidol has high hydrophobicity; thus, nerolidol might be used in drug delivery systems, such as lipid nanoparticles to treat leishmaniasis.

## Introduction

Leishmaniasis is caused by protozoan parasites from more than 20 *Leishmania* species, and it is transmitted to humans via the bite of infected female phlebotomine sandflies [1]. The clinical spectrum of leishmaniasis manifests in three forms: cutaneous (the most common), visceral (the most serious form, which is fatal if left untreated) and mucocutaneous. A recent report from the World Health Organization (WHO) [1] indicated that as many as 12 million people are currently infected, with approximately 1.3 million estimated new cases occurring every year and an estimated 20,000 to 30,000 deaths caused by these parasites annually.

Pentavalent antimonials are the first-line drugs for treating the cutaneous form of American tegumentary leishmaniasis [2], although they are characterized by high toxicity and limited efficacy [3]. Amphotericin B is more toxic and difficult to administer, but it has replaced antimonials for treating visceral leishmaniasis in some areas of the Bihar State of India where more than 60% of newly diagnosed cases do not respond to antimonials due to resistance [3]–[5]. Liposomal amphotericin B is currently considered the first-line treatment for visceral leishmaniasis [4], especially after the price reduction of 90% that was announced recently by WHO [1]. Miltefosine was the first effective oral drug approved for the treatment of visceral and cutaneous leishmaniasis. This drug was registered in India (2002), Germany (2004) and Colombia (2005) and has shown high cure rates in the treatment of visceral [4], cutaneous [5] and mucocutaneous leishmaniasis [6], but its teratogenicity prevents its use in pregnant women [4], [6]. Leishmaniasis is a poverty-related disease, and the major concerns of WHO are reducing the cost of medications and preventing the development of drug resistance [1]. In this context, the search for new active compounds against *Leishmania* from the global biodiversity represents a promising opportunity for discovering new drugs [7], [8].

Terpenes are constituents of the essential oils of various plants and flowers, which only contain carbon, hydrogen and oxygen atoms. Physiologically, terpenes function mainly as chemoattractants or chemorepellents and are largely responsible for the characteristic fragrance of many plants [9], [10]. Several dietary monoterpenes have shown antitumor activity and are effective in the chemoprevention of and chemotherapy for cancer [10]–[16]. In addition, essential oils or their terpenes have also shown antibacterial, antifungal, antiparasitic, antiviral, anti-allergenic, and anti-inflammatory activity [15], [16]. The monoterpene terpinen-4-ol was reported to show antifungal activity [17], and both the monoterpene limonene [18] and the sesquiterpene nerolidol [19] have been reported to show antileishmanial activity.

Electron paramagnetic resonance (EPR) spectroscopy using spin labels has been employed to investigate the mechanisms underlying the acceleration of skin permeation by monoterpenes [20]–[23]. The intercellular lipid matrix of the stratum corneum, the outermost skin layer, which represents the major permeability barrier of the skin, becomes more fluid in the presence of the monoterpenes L-menthol and 1,8-cineole [20], [21]. In addition, treatment with monoterpenes increases the partition coefficient of the small water-soluble spin labels TEMPO and DTBN into stratum corneum membranes [22], [23]. EPR spectroscopy was used recently to demonstrate that miltefosine causes remarkable increases in the membrane fluidity of the stratum corneum tissue [24] and also induces dramatic increases in the lipid and protein dynamics of erythrocyte [25] and *L. amazonensis* membranes [26]. In this study, the cytotoxicity effects of three monoterpenes and one sesquiterpene on *L. amazonensis* promastigotes were compared with the dynamic changes in the parasite membrane. Our results from several experimental conditions demonstrate that the cytotoxicity effect and membrane alteration caused by these terpenes follow similar concentration dependencies.

## Materials and Methods

### Chemicals

**Figure 1:**
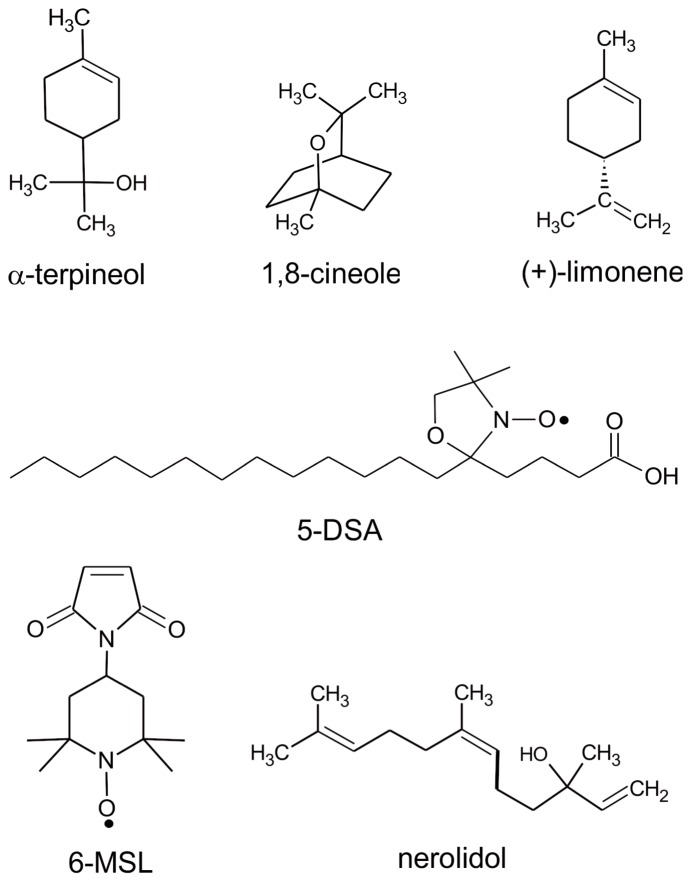
Chemical structures. The four terpenes and the spin labels 5-DSA and 6-MSL used in this study.

The spin labels 5-doxyl stearic acid (5-DSA) and 4-maleimido-1-oxyl-2,2,6,6-tetramethylpiperidine (6-MSL) (Fig. 1) were purchased from Sigma-Aldrich (St. Louis, USA), whereas the terpenes (Fig. 1) were purchased from Acros Organics (Geel, Belgium).

### Parasites

Promastigotes of *L. (L.) amazonensis* (MHOM/BR/75/Josefa) reference strains were grown in 24-well microtiter plates containing Grace's insect medium (Sigma-Aldrich), supplemented with 20% heat-inactivated fetal calf serum (FCS), 2 mM L-glutamine, 100 U/mL penicillin and 100 µg/mL streptomycin (Sigma-Aldrich), as previously described [27].

### Spin labeling and treatment with terpenes

To incorporate the lipid spin label into the *Leishmania* membranes, a film of the 5-DSA was first generated on the bottom of a test tube, as in previous work [26]. A 1 µL aliquot of a stock solution of the spin label 5-DSA in ethanol (4 mg/mL) was transferred to a glass tube and, after solvent evaporation, 50 µL of a *Leishmania* suspension containing 10^8^ cells in PBS was added to the spin-label film, followed by gentle agitation. The spin labeling of *L.* membrane proteins was performed through incubation for 2 h at 26°C after the addition of an excess of the thiol-specific spin label 6-MSL. To remove the free spin labels, the *Leishmania* suspension was centrifuged (5,000×g, 4°C) for 15 min and then resuspended in PBS; this procedure was repeated 8 times. Following spin labeling, terpenes dissolved in 33% ethanol (v/v) were added to *Leishmania* cells, and the sample was gently stirred. The maximum percentage of ethanol used to deliver the highest drug concentrations in the samples was 4%, and this amount did not change the EPR spectra of the spin labels when compared with the control sample without ethanol. The terpene concentrations used in the EPR experiments ranged from 0.12 to 14.5×10^9^ terpene molecules/cell (for concentrations above 2.4×10^9^ terpene molecules/cell, the terpenes were applied without dilution in ethanol). After labeling and treatment, the samples were transferred to 1-mm i.d. capillary tubes, which were sealed using a flame, for the EPR measurements.

### Membrane disruption measurements

*L. amazonensis* promastigotes were treated with terpenes as described above for the EPR experiments. After treatment (1–5 min), the samples were centrifuged at 25,000×g for 15 min at room temperature and the protein content in the supernatant was measured using a commercial kit (Sigma) based on the bicinchoninic acid (BCA) reaction.

### *In vitro* antiproliferative activity assays

Parasites at four chosen concentrations (5.0×10^6^, 1.0×10^7^, 1.0×10^8^ and 2.0×10^9^ cells/mL) were incubated in culture medium supplemented with 10% FCS for 24 h in 96-well culture dishes (Corning Life Sciences, Corning, USA) containing the culture medium with increasing concentrations of the studied terpenes, which were initially diluted in ethanol to 33% (v/v). In this test, the maximum ethanol volume in the medium was less than 1%, which caused no detectable parasite toxicity. Cell viability was assessed by measuring the cleavage of 3-(4,5-dimethylthiazol-2-yl)-2,5-diphenyl tetrazolium bromide (MTT; Sigma-Aldrich) by metabolically active cells, as described previously [28]. Briefly, the parasites were incubated in a solution containing 5 mg/mL MTT for 3 h at 26°C and the absorbance was read at 550 nm using a T80+ spectrophotometer from PG Instruments (Wibtoft, England). Measurements were performed in triplicate for each treatment, and the obtained values were used to calculate the mean percentage of viable cells relative to the control. The 50% inhibitory concentration (IC_50_) was then determined using sigmoidal fitting (Boltzmann function) of the concentration–response curve. For some experiments, the terpene treatment was performed in the same manner as in the EPR experiments, and the antiproliferative activity was determined by adding MTT after an incubation period of 5 minutes.

### EPR spectroscopy

A Bruker ESP 300 spectrometer (Rheinstetten, Germany) equipped with an ER4102ST resonator was used to perform the EPR measurements. The instrument parameters were as follows: microwave power, 2 mW; modulation frequency, 100 KHz; modulation amplitude, 1.0 G; magnetic field scan, 100 G; sweep time, 168 s; and sample temperature, 25°C. EPR spectra simulations were conducted using the nonlinear least-squares (NLLS) fitting program [29]. For the spectral calculations, the NLLS program includes the magnetic g- and A-tensors, which are expressed in a system of Cartesian axes that are fixed on the spin-labeled molecule. To reduce the number of parameters in the fitting and simplify the simulation, the average rotational diffusion rate (R_bar_) was calculated by the fitting program based on the relationship R_bar_ = (R_per_.2R_par_)^1/3^, where R_per_ and R_par_ are the perpendicular and parallel components of the rotational diffusion, respectively, relative to the principal axis of symmetry of the spin-labeled molecule, considering axial symmetry. R_bar_ was converted to the rotational correlation time (τ_c_) parameter using the relationship τ_c_ = 1/6 R_bar_ [29]. Similar to previous studies [24], [30], the spectra were simulated using a model including one or two spectral components. Through an overall analysis of the spectra in this work with the NLLS program, the principal values of the g- and A-tensors were optimized and, once determined, were fixed in the simulation of all spectra. The values used for components 1 and 2 were as follows: g_xx_(1) = 2.0088, g_yy_(1) = 2.0058, g_zz_(1) = 2.0028, A_xx_(1) = 6.6 G, A_yy_(1) = 6.5 G, A_zz_(1) = 33.0 G, g_xx_(2) = 2.0088, g_yy_(2) = 2.0058, g_zz_(2) = 2.0028, A_xx_(2) = 5.5 G, A_yy_(2) = 5.5 G and A_zz_(2) = 30.8 G.

### Statistical analysis

All data presented are expressed as the mean ± S.D. from at least three independent experiments. Data were compared via one-way analysis of variance (ANOVA) followed by Tukey's multiple range test for statistically significant differences at P<0.05.

## Results

### Terpenes increase the lipid dynamics in *Leishmania* membranes

**Figure 2:**
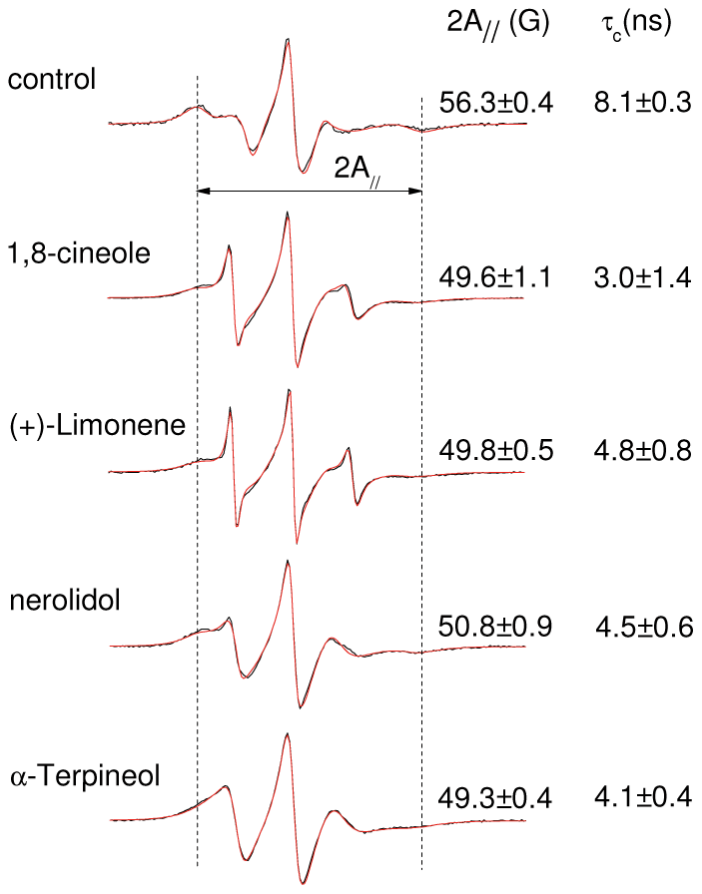
EPR spectra of spin-labeled *Leishmania* plasma membrane. Experimental (black lines) and best-fit (red lines) EPR spectra of 5-DSA in the plasma membranes of *Leishmania amazonensis* promastigotes from untreated and terpene-treated samples (4.8×10^9^ terpenes/cell). The best-fit spectra were obtained with the NLLS fitting, which provided the average rotational correlation time, τ_c_, for each spectrum. Another parameter that is measured directly in the EPR spectrum, 2A_//_, is indicated by the vertical lines in the figure. The mean and S.D. of τ_c_ and 2A_//_ are indicated. For both parameters, all terpene-treated samples were significantly different from the control sample (P<0.01).

The EPR spectra of the spin-labeled lipid 5-DSA (Fig. 1) that was incorporated into the plasma membranes of *L.* promastigotes are shown in Fig. 2 for samples with and without terpene treatment. The localization of the spin label in the *Leishmania* plasma membrane and the uniformity of the probe distribution throughout the membrane are ensured by the EPR spectra, which do not have free signal characteristics of spin labels outside the cell or dipolar interactions that would be caused by the proximity between the probes. If the spin label enters the cell, it quickly loses its signal because of nitroxide reduction by cytoplasmic agents such as ascorbic acid, Fe(II) and thiol groups. Spin-labeled lipids have been successfully incorporated into stratum corneum membranes [24] and the cellular membranes of coffee root-tip segments [31], *Trypanosoma cruz*i protozoans [32] and fibroblasts [30]. The spectra for the terpene-treated samples were characterized by the presence of two resolved spectral components, that is, two populations of spin labels with different mobility states. Two-component EPR spectra are commonly observed for spin labels in simple bilayers [33], and terpenes clearly favor the formation of the more mobile component [20], [21]. The interpretation is that the more mobile component is provided by a fraction of spin labels that are more deeply inserted into the hydrophobic core of the membrane [20], [21]. Fits of the spectra to a two-state model were used to determine the relative population and motional parameter of each spectral component. As discussed in previous works [24], [30], the average value of the motion parameter τ_c_ was calculated from parameters generated by the simulations using the following equation:
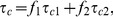
(1)where f_1_ and f_2_ represent the relative fractions of the spin label in the less (1) and more (2) mobile components, respectively, and τ_c1_ and τ_c2_ are the respective rotational correlation times. The treatments with terpenes caused large reductions in the τ_c_ values, indicating dramatic increases in the *Leishmania* membrane fluidity.

### Cytotoxicity measurements

EPR experiments require *Leishmania* samples with high cell concentrations, and in this work, we used 2×10^9^ cells/mL. However, we note that the molar terpene concentration required to change the membrane fluidity increases as the parasite concentration used in the experiment increases. The terpene concentration used to obtain the spectra shown in Fig. 2 was 4.8×10^9^ terpene molecules/cell, which corresponds to 16 mM in the cell suspension. Because the reported IC_50_ values of the active compounds in *Leishmania* generally fall in the micromolar range, this terpene concentration is too high. In a previous work [26], it was found that the IC_50_ of miltefosine in *L. amazonensis* promastigotes increases as the cell concentration used in the MTT colorimetric assay increases. Thus, we decided to measure the IC_50_ of terpenes using four distinct *Leishmania* concentrations (Table 1). When expressed in molar concentration, the IC_50_ increased as the assay cell concentration increased and tended to decrease when expressed in the number of terpene molecules per cell. For 2×10^9^ cells/mL, the experiment was also performed with an incubation period of only 5 minutes (as in the EPR experiments) before the addition of MTT. The IC_50_ values found for the 24 h and 5 min of incubations did not differ significantly. Terpenes are hydrophobic molecules and their incorporation into membranes is rapid when using samples with a high cell concentration. This last experiment indicated that the activity of each terpene against *Leishmania* does not require a long incubation period.

**Table 1:** Effect of terpenes against *Leishmania amazonensis* promastigotes (MTT assay).

Terpene	Incubation^a^	IC_50_ (µM)	IC_50_(10^9^terpenes/cell)
5.0×10^6^ Cells/mL			
nerolidol	24 h	7.9±0.8 (A)^b^	0.95±0.09 (A)
(+)-limonene	24 h	549±169 (B)	66±20 (B)
α-terpineol	24 h	678±59 (B)	83±6 (B)
1,8-cineole	24 h	4697±1258 (C)	565±151 (C)
1.0×10^7^ Cells/mL			
nerolidol	24 h	9.5±0.9 (A)	0.57±0.06 (A)
(+)-limonene	24 h	833±146 (B)	50±9 (B)
α-terpineol	24 h	1115±374 (B)	62±18 (B)
1,8-cineole	24 h	6885±1011 (C)	400±50 (C)
1.0×10^8^ Cells/mL			
nerolidol	24 h	29.2±5.2 (A)	0.17±0.03 (A)
(+)-limonene	24 h	1920±130 (B)	11.6±0.8 (B)
α-terpineol	24 h	2203±395 (B)	13.3±2.4 (B)
1,8-cineole	24 h	9637±780 (C)	58±5 (C)
2.0×10^9^ Cells/mL			
nerolidol	24 h	1109±148 (A)	0.33±0.04 (A)
	5 min	1049±280 (A)	0.32±0.08 (A)
(+)-limonene	24 h	4803±315 (B)	1.42±0.09 (B)
	5 min	5322±559 (B)	1.57±0.19 (B)
α-terpineol	24 h	2950±499 (C)	0.89±0.15 (C)
	5 min	3402±449 (C)	1.02±0.14 (C)
1,8-cineole	5 min	8048±848 (D)	2.42±0.25 (D)

a Period of incubation after treatment with terpenes and before the MTT addition.

b Statistical analysis: The data of the same column and cell concentration were compared statistically with P<0.05. Means of the same column and cell concentration that are not listed with capital letters in common are statistically different. For all terpenes the means for cell concentrations of 5.0×10^6^ cells/mL and 2.0×10^9^ cells/mL are significantly different (P<0.05).

When calculating the molar concentration of a molecule with a high affinity for the membrane in a cell suspension, inaccuracies may occur in the calculated concentration because a considerable fraction of the molecules enters the membranes, thus reducing the molar concentration in the aqueous medium. To examine this issue in more detail, let us consider a cell suspension with n_w_ moles of the test molecule in the aqueous medium and n_m_ moles in the membrane interior. The calculated molar concentration (c_cal_) will be:
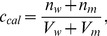
(2)where V_w_ is the water volume and V_m_ is the membrane volume in the suspension. Rewriting eq. 2 in terms of the molar concentrations in the membrane, c_m_, and in the aqueous medium, c_w_, and introducing the partition coefficient K, where K = c_m_/c_w_, we have:
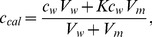
(3)or
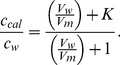
(4)


**Figure 3:**
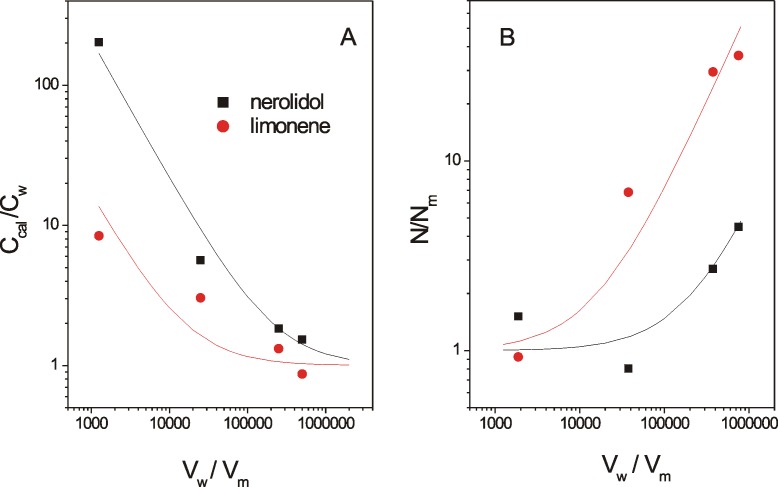
Theoretical and experimental IC_50_ dependencies on the cell concentration. (A) Theoretical curves calculated from eq. 4 (see text for details) for the molar concentration ratio c_cal_/c_w_ in comparison with the IC_50_ values shown in Table 1 for different cell concentrations of nerolidol (square) and (+)-limonene (circle) as a function of V_w_/V_m_. (B) Theoretical curves calculated from eq. 8 for the ratio N/N_m_ compared with the IC_50_ of nerolidol and (+)-limonene versus V_w_/V_m_.

If V_w_>>V_m_, then eq. 4 indicates that c_cal_ = c_w_, i.e., the calculated concentration is accurate for well-diluted systems. Fig. 3A shows a plot of the relative IC_50_ values expressed in molar concentrations for nerolidol and limonene (Table 1) and the best-fit curves of c_cal_/c_w_ from eq. 4; for these curves, the octanol-water partition coefficients of these terpenes were used as estimates of their K values. Reports have indicated that log P_o/w_ = 5.33 for nerolidol and log P_o/w_ = 4.23 for (+)-limonene (Bio-Pesticides DataBase, http://sitem.herts.ac.uk/aeru/bpdb/2114.htm); thus, we used K values of 2.14×10^5^ (nerolidol) and 1.70×10^4^ (limonene). The four IC_50_ values of each terpene were multiplied by convenient constants to facilitate a comparison with their theoretical curves. The fitting between the experimental and theoretical data provided a ratio of V_w_/V_m_ = 5.0×10^5^ for the lowest cell concentration used (5×10^6^ cells/mL), and for the other cell concentrations, the volumetric ratios were calculated based on this relationship. When c_cal_/c_w_ approaches 1 in the plot, the system can be considered dilute. Note that for the adjusted experimental curve of limonene, the experimental point corresponding to the lowest cell concentration used (5×10^6^ cells/mL) is close to 1, which is consistent with a dilute system for this cell concentration; however, in the case of nerolidol with a larger K value, this cell concentration cannot be considered sufficiently dilute (c_cal_/c_w_∼2).

Because the calculated IC_50_ values are inaccurate for larger cell concentrations, such as those used in the EPR experiments (2×10^9^/mL), as indicated in Table 1, we also expressed the IC_50_ in terpene molecules/cell. However, the IC_50_ expressed in these units also depended on the cell concentration. In this case, the calculation is more accurate for high cell concentrations because for a lower cell concentration, a majority of the test molecules will be diluted in the aqueous medium outside of the cells. This calculation can be performed using the following equation:
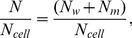
(5)where N, N_w_ and N_m_ represent the numbers of molecules in the suspension and in the aqueous and membrane media, respectively, and N_cell_ represents the number of cells in the suspension. Rewriting eq. 5 in terms of molar concentrations and Avogadro's number (N_A_) one can derive the following expressions:
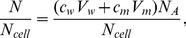
(6)or
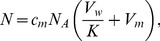
(7)and
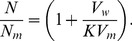
(8)


If V_w_ is very small, then in eq. 8, N = N_m_, i.e., almost all test molecules are in the cell membranes, indicating accurate calculations for suspensions with high cell concentrations. In this calculation, the number of molecules entering inside the cell was neglected (we estimate to be less than 1% relative to the number entering the membrane). The graph in Fig. 3B shows the theoretical curves of N/N_m_ given by eq. 8 using log K = 5.33 for nerolidol and log K = 4.23 for limonene. In this case, the experimental data for these two terpenes (Table 1) fit better when using the ratio V_w_/V_m_ = 7.4×10^5^ for 5×10^6^ cells/mL. From the plot of nerolidol, the value of N/N_m_ approaches 1 for the two largest tested cell concentrations (2×10^9^ and 1×10^8^ cells/mL), suggesting more accurate calculations for these two cell concentrations. In contrast, for limonene, N/N_m_ was ∼1 for 2×10^9^ cells/mL but was ∼10 for 1×10^8^ cells/mL, suggesting that the latter cell concentration was not sufficiently high for an accurate calculation of the terpene molecules/cell for this terpene.

### Cytotoxicity versus membrane fluidity

**Figure 4:**
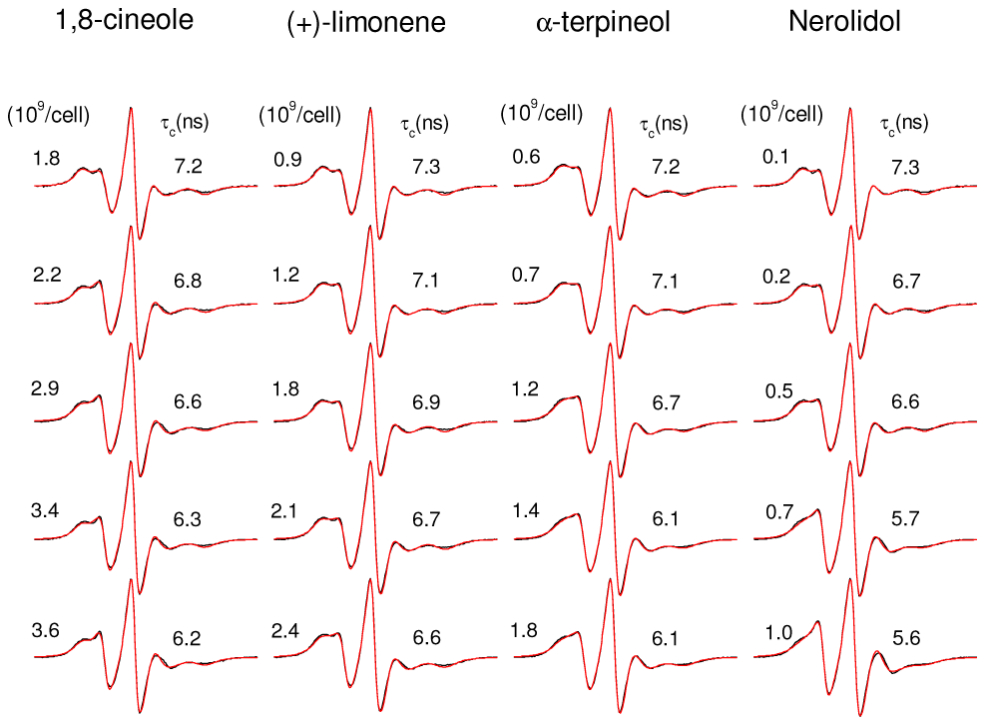
EPR spectra of spin-labeled *Leishmania* plasma membrane. Experimental (black lines) and best-fit (red lines) EPR spectra of 5-DSA in the plasma membranes of *Leishmania amazonensis* promastigotes for samples treated with terpenes at several concentrations around their IC_50_ (two concentrations below and three above the IC_50_). The EPR spectra are representative of three independent experiments and the means and S.D. of the rotational correlation time (τ_c_) obtained from the NLLS fitting are indicated. The spectra of the control samples (without treatment) were similar to those already shown in Fig. 2 (τ_c_ = 8.1±0.2 ns).

**Figure 5:**
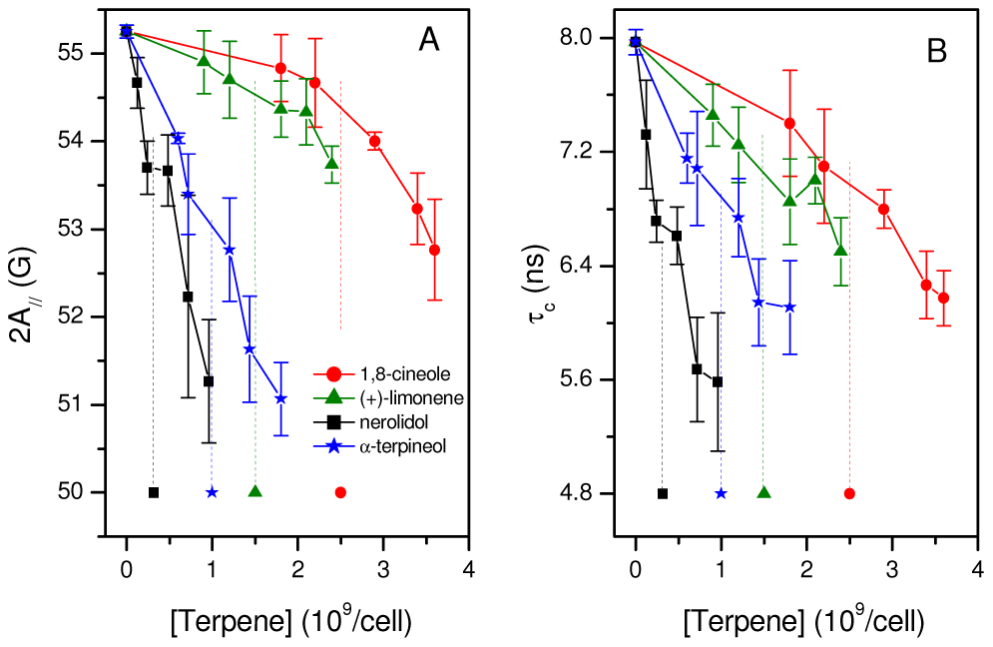
Effect of terpenes on the dynamics of the *Leishmania* plasma membrane. EPR parameters 2A_//_ (panel A) and rotational correlation time, τ_c_, (panel B) of the spin label 5-DSA in the membranes of *Leishmania amazonensis* promastigotes as a function of the terpene concentration used in the treatment. The IC_50_ values of the terpenes are represented by single points at the bottom of each curve and dotted lines. The estimated experimental errors for 2A_//_ and τ_c_ are 0.5 G and 0.2 ns, respectively.

To examine whether there is a relationship between cytotoxicity and alteration in membrane fluidity, we performed the terpene treatment of *Leishmania* using the same experimental conditions for two comparative experiments. After 5 minutes of incubation, either the MTT was added for the cytotoxicity test or the spin label 5-DSA was incorporated into the membranes for EPR measurements. The IC_50_ values obtained have already been presented in Table 1 (incubation of 5 min), and the EPR spectra are shown in Fig. 4 for two concentrations below and three above the IC_50_ of each terpene studied. The plots of the EPR parameters 2A_//_ and τ_c_ are shown in Fig. 5. These parameters clearly decrease for terpene concentrations in the IC_50_ region of each terpene, showing a clear correlation between the cytotoxicity and the increase in the *L.* membrane fluidity.

### Effect of terpenes on *Leishmania* membrane proteins

**Figure 6:**
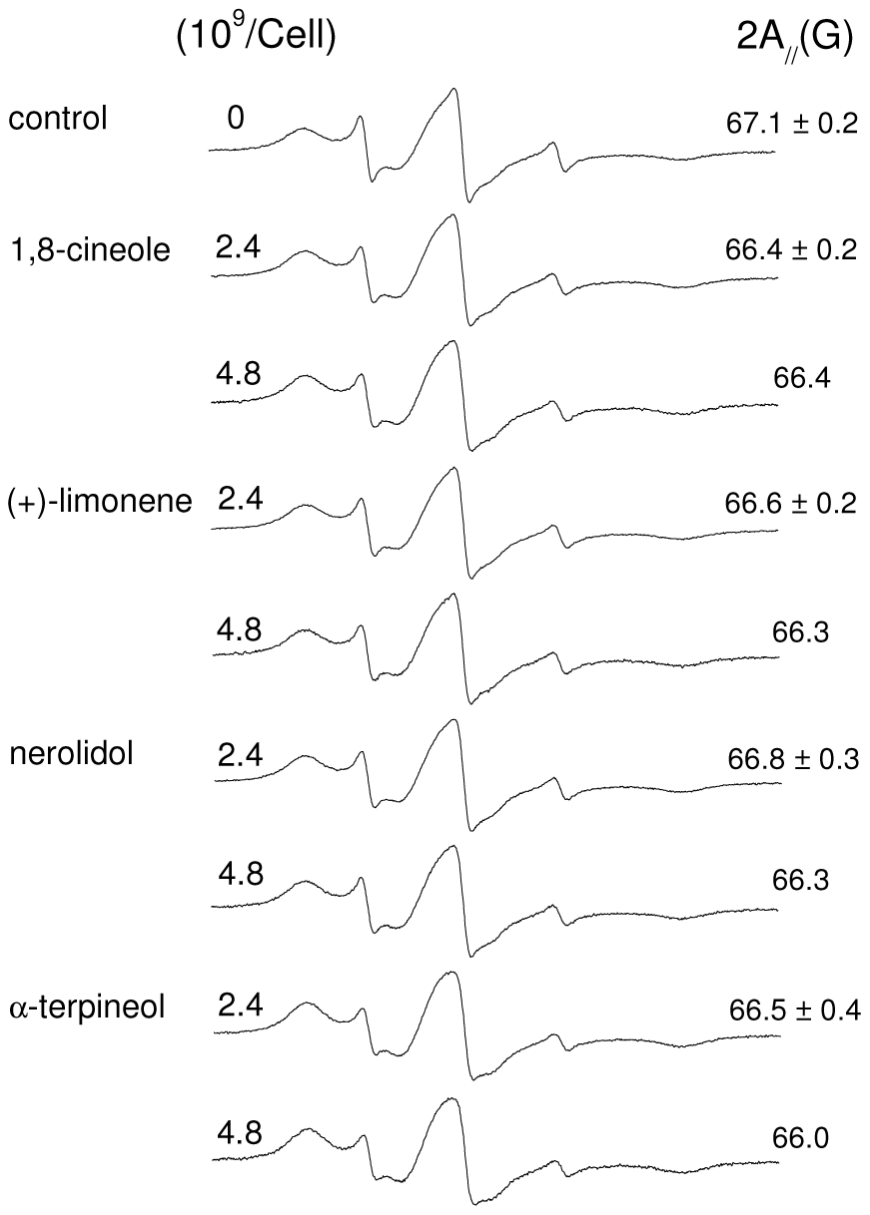
EPR spectra of spin-labeled membrane proteins of *Leishmania* for untreated and terpene-treated samples. The 2A_//_-parameter values are indicated.

A previous study demonstrated that miltefosine has a strong effect on the membrane-bound proteins of *L. amazonensis* promastigotes when examined by a maleimide-derivative spin label covalently bound to the sulfhydryl groups of the parasite plasma membrane [26]. To examine whether terpenes alter membrane protein dynamics, we performed experiments under conditions identical to those of the previous work. The EPR spectra of the maleimide spin label 6-MSL bound to the *L.* membrane are shown in Fig. 6 for untreated and terpene-treated samples. Interestingly, despite the variety of membrane proteins, the EPR spectrum was similar to that of BSA spin labeled at its single sulfhydryl group [34], suggesting that the sulfhydryl groups of different proteins have a similar local structure. Based on the intensity of the EPR spectra, it is possible to estimate the amount of spin-labeled sulfhydryl groups to be approximately 10^7^/cell, which is similar to the amount found for erythrocyte membranes [25]. The tested terpene concentrations were very high, and the smallest concentration (4.8×10^9^ terpene molecules/cell) was approximately twice the IC_50_ of the least active of the four terpenes (1,8-cineole). Only small decreases in the values of 2A_//_ were observed; the values for the samples treated with 4.8×10^9^ terpene molecules/cell were not significantly different from those of the control sample. For the highest terpene concentration, the decreases in 2A_//_ were only slightly higher than the estimated experimental error. As discussed in a previous study [26], the 2A_//_ parameter reflects the backbone protein dynamics, and our results suggest that terpenes cause only small increases in the protein dynamics of the *L.* membrane.

### Terpenes induce lysis of *Leishmania*

**Figure 7:**
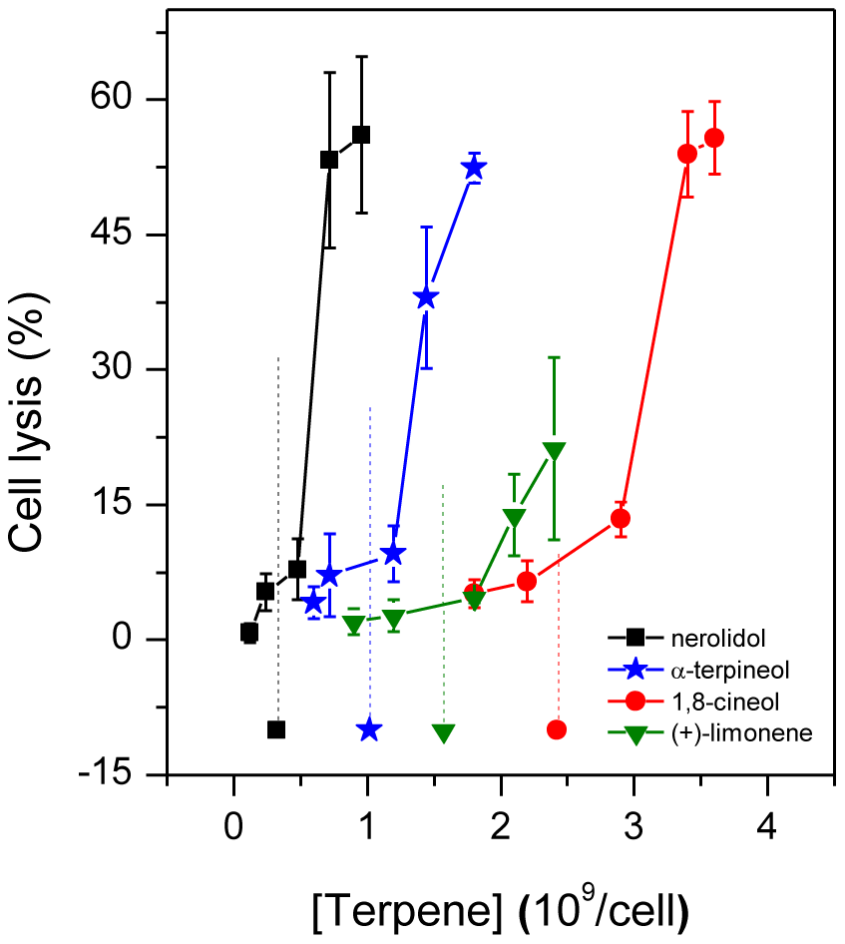
Percentage of cell lysis in samples of *Leishmania amazonensis* promastigotes treated with terpenes. The IC_50_ of each terpene is represented by its corresponding symbol and a vertical line.

Cell lysis was also observed under the range of terpene concentrations that alter *L.* membrane fluidity. Terpene-treated *Leishmania* samples prepared under the same experimental conditions as used for the EPR measurements and cytotoxicity assay (cell concentration of 2×10^9^ cells/mL and incubation period of 5 minutes) were centrifuged to measure the cell lysis (Fig. 7). At the terpene concentration corresponding to the IC_50_ of each terpene, the percentages of cell lysis observed for the nerolidol, α-terpineol, (+)-limonene and 1,8-cineole treatments were approximately 6, 8.5, 4 and 9%, respectively. At the highest measured terpene concentration, the cell lysis reached ∼20% for limonene and ∼50% for the other terpenes.

## Discussion

EPR spectroscopy using lipid spin labels has been widely used to assess the cell membrane fluidity of erythrocytes in suspension for hematocrit values that generally range from 20–50% (∼2.2–5.5×10^9^ RBCs/mL), and the drug concentration that causes a change in membrane fluidity generally falls in the millimolar range [25]. This condition is physiologically relevant because human blood has a typical hematocrit of 42% and ∼5×10^9^ erythrocytes/mL. To measure the antiproliferative activity of *Leishmania* using the MTT colorimetric assay, a lower cell concentration of approximately 5×10^6^ cells/mL is generally used. For culture medium supplemented with fetal calf serum, the albumin/cell ratio in the suspension increases as the system is diluted, and for hydrophobic drugs, the amount of drug sequestered by albumin becomes larger, thus requiring more time for incorporation of the drug into the cell membrane. A previous study [26] demonstrated that using 20% instead of 10% fetal calf serum in the MTT assay significantly increased the IC_50_ of miltefosine against *L. amazonensis* promastigotes because a higher miltefosine concentration was retained in the albumin. The same study also showed that the miltefosine IC_50_ was significantly higher when an incubation period of 1 h was used instead of 24 h. However, using a method for rapidly incorporating miltefosine into the membranes of *Leishmania*, cytotoxicity effects were observed without any incubation [26].

The partition theory indicates that the permeability constant for the transport of a molecule across a membrane is proportional to the partition coefficient K [22], [23]. The coefficient log P_O/W_ is commonly used as an estimate of log K because it is easier to measure. In suspensions with small numbers of cells, the fraction of molecules that enters the membranes can be neglected, and the system can be considered homogenous. In this case, the calculated molar concentration of drug in the suspension is accurate and represents the exact concentration of the drug in the aqueous phase. However, for larger numbers of cells, the fraction of drug that enters the membrane cannot be neglected, the system is no longer homogeneous, and the drug concentration in the aqueous medium is lower than the calculated concentration. As the partition coefficient, given by K = c_m_/c_w_, is a constant, a lower concentration in the aqueous medium also leads to a reduction in the membrane drug concentration. In this study, we demonstrated that the IC_50_ of four terpenes in *L. amazonensis* promastigotes increases as the cell concentration used in the MTT assay increases. This effect was attributed to a miscalculation of the molar concentration to hydrophobic drugs in cell suspension, and the IC_50_ values obtained were consistent with our theoretical prediction (Fig. 3A). In the case of nerolidol, which had the highest log P_O/W_ of the studied terpenes, the lowest cell concentration used in our experiments (Fig. 3A) cannot be considered sufficiently dilute, suggesting that the IC_50_ of 7.9 µM found using 5×10^6^ cells/mL could be even smaller for lower cell concentration (5.6 µM). A miltefosine IC_50_ of 59.3 µM has been reported for 1.0×10^8^ cells/mL [26], and this value is comparable to the nerolidol IC_50_ of 29.2 µM found here (Table 1) for the same parasite and experimental conditions; however, the IC_50_ values of (+)-limonene, α-terpineol and 1,8-cineole were much larger than those of miltefosine and nerolidol (Table 1).

For samples with a high concentration of cells, it is more convenient to express the IC_50_ of hydrophobic drugs as the number of molecules per cell. In this calculation, the number of molecules that remain in the aqueous phase outside the cell is neglected. The terpene-IC_50_ measurements (Table 1) and the theoretical prediction (Fig. 3B) indicated that only the 2×10^9^ cells/mL concentration was sufficiently concentrated for an accurate calculation. At this cell concentration, the nerolidol IC_50_ was 0.32×10^9^ terpene molecules/cell, which was similar to that of miltefosine under the same experimental conditions [26]. To obtain the best fit of the IC_50_ for nerolidol and (+)-limonene to the theoretical curves given by eqs. 4 and 8 (Figs. 3A and 3B), V_w_/V_m_ (volume water/volume membrane) ratios of 5.0×10^5^ and 7.4×10^5^, respectively, were assumed for the samples with 5×10^6^ cells/mL. If we average these volumetric ratios in the suspension (6.2×10^5^), we can calculate that there are 3.2×10^−19^ m^3^ of membrane per *Leishmania*. Assuming a *Leishmania* membrane thickness of 8×10^−9^ m and only considering the plasma membrane, one can calculate the superficial area of the parasite to be 4.0×10^−11^ m^2^. Assuming a spherical shape for *Leishmania*, its diameter would be 3.6 µm. *L. amazonensis* promastigotes are spindle-shaped cells that are approximately 8 µm in length and 2 µm in diameter [35]. This relatively good estimate of the parasite size, based on the IC_50_ of limonene and nerolidol, indicates consistency between our MTT assay data for high and low cell concentrations and a 24 h-incubation period.

Cytotoxicity measurements at high cell concentrations were necessary to show that at concentrations close to their IC_50_, the studied terpenes cause important increases in the parasite membrane fluidity and small amounts of cell lysis ranging from 4 to 9% (Fig. 7). Furthermore, when the cytotoxicity was measured using 2×10^9^ cells/mL, the results for 24 h and 5 min of incubation were not significantly different, suggesting that the incorporation of terpenes into the *L.* membranes is very fast at high cell concentrations, most likely due to the lower volume of the aqueous phase and the smaller quantity of albumin in the suspension. These results indicate that the cytotoxicity of terpenes against *L.* promastigotes is associated with an increase in the plasma membrane fluidity and with membrane rupture.

Other studies have reported IC_50_ values for limonene [18] and nerolidol [19] against *L. amazonensis* promastigotes using the MTT assay. Limonene induced antiproliferative effects with an IC_50_ of 252 µM for 1.0×10^6^ cells/mL, which is lower than that found in this work (549 µM, Table 1) for a higher cell concentration (5.0×10^6^ cells/mL). However, in the case of nerolidol, the IC_50_ found here (7.9 µM, Table 1) was smaller than the reported value [19]. In a study comparing the cytotoxicity in fibroblast cells and the hemolytic potential of seven monoterpenes and the sesquiterpene nerolidol, the highest cytotoxic and hemolytic potentials were found for nerolidol and α-terpineol, whereas the lowest were found for 1,8-cineole and limonene [30].

Our data can be compared with the reported data from studies on the *in vitro* antiproliferative activity of terpenes in other parasites and cancer cells. Geraniol and limonene showed antiplasmodial activities against *Plasmodium falciparum* with IC_50_ values of 52 and 66 µM, respectively, whereas the control drugs chloroquine and artemisinin showed IC_50_ values of 291 nM and 7 nM, respectively [36]. These last two concentrations are too low to cause a change in the plasma membrane fluidity that is detectable by EPR spectroscopy; however, the first two concentrations are sufficiently high to change the membrane fluidity. In this methodology, a spin-labeled molecule is used for approximately 150 natural membrane lipids. Thus, to detect any changes in membrane fluidity, the environment of most spin labels must change, requiring a higher concentration of the drug, usually in the micromolar range. Perillyl alcohol inhibited the proliferation of human non-small cell lung cancer cells (A549 and H520) with IC_50_ values greater than 1 mM, inducing cell cycle arrest and apoptosis with increases in the expression of Bcl_2_, bax and p21 and in the caspase-3 activity [37]. Tea tree oil and its major active terpene component, terpinen-4-ol, demonstrated antiproliferative activity against two murine tumor cell lines, AE17 mesothelioma and B16 melanoma, with IC_50_ values equal to or greater than 0.01% (∼588 µM), and they induced necrotic cell death and cell cycle arrest in both tumor cell lines [38]. Miltefosine is also able to increase membrane fluidity and induce cell lysis in *L. amazonensis* promastigotes at concentrations close to its IC_50_ [26]. Interestingly, miltefosine has been shown to reduce the mitochondrial membrane potential and inhibit cytochrome c oxidase, changes that are linked to the apoptosis-like cell death [39]. Our results suggest that further studies should be performed to examine whether the various reported mechanisms for the antiproliferative effect of terpenes are triggered by a primary attack on the cell membrane.

In conclusion, our present findings demonstrate that the IC_50_ values of the terpenes nerolidol, (+)-limonene, α-terpineol and 1,8-cineole against *L. amazonensis* promastigotes increase as the cell concentration used in the MTT assay increases and that these increases follow our theoretical prediction for their dependencies. This evidence implies that the usual method used to calculate the IC_50_ for cytotoxicity assays does not apply in the case of hydrophobic drugs and that corrections based on the material discussed in this work are required. Terpenes caused increases in the *L.* membrane fluidity, and this effect was observed for each terpene at concentrations similar to their IC_50_ values. Furthermore, the terpenes caused a small amount of cell lysis ranging from 4 to 9% at concentrations close to their respective IC_50_. At the high cell concentrations used in the assay (2×10^9^ parasites/mL), the incorporation of terpene into the cell membrane becomes fast, and the IC_50_ values observed for a 24 h-incubation period were not significantly different from those found with only 5 minutes of incubation, suggesting that the mechanism of action of terpenes is associated with the attack on the plasma membrane of the parasite. As demonstrated by the spin-label EPR data of the membrane-bound proteins, the presence of these terpenes at cytotoxic concentrations did not significantly alter the dynamics of the parasite membrane proteins, suggesting that the action of terpenes is restricted to the lipid component of the membrane. A mechanism of action based on an attack on the *Leishmania* plasma membrane is consistent with the reported broad spectrum of terpene activity against protozoa, pathogenic fungi and tumor cells. Terpenes are considered to be potent skin permeation enhancers, and those with leishmanicidal activity would be very promising candidates for integrating nanocarriers for the treatment of cutaneous leishmaniasis.
